# Supplementary material for: Crowdsourced analysis of fungal growth and branching on microfluidic platforms
Source: PLoS One. 2021 Sep 29;16(9):e0257823. doi: 10.1371/journal.pone.0257823 (PMC8480888; doi:10.1371/journal.pone.0257823)
Supplement: S1 File — (DOCX) [file pone.0257823.s001.docx]

1.) Using a P200 pipette, prime the device by entering the central chamber and pushing the appropriate growth media through the device. Look for media escaping from the outside edges of the PDMS device to know when media has made it through all the features. If this first priming step does not exhibit media droplets outside the device, a second priming of the device may be attempted after Step 2.


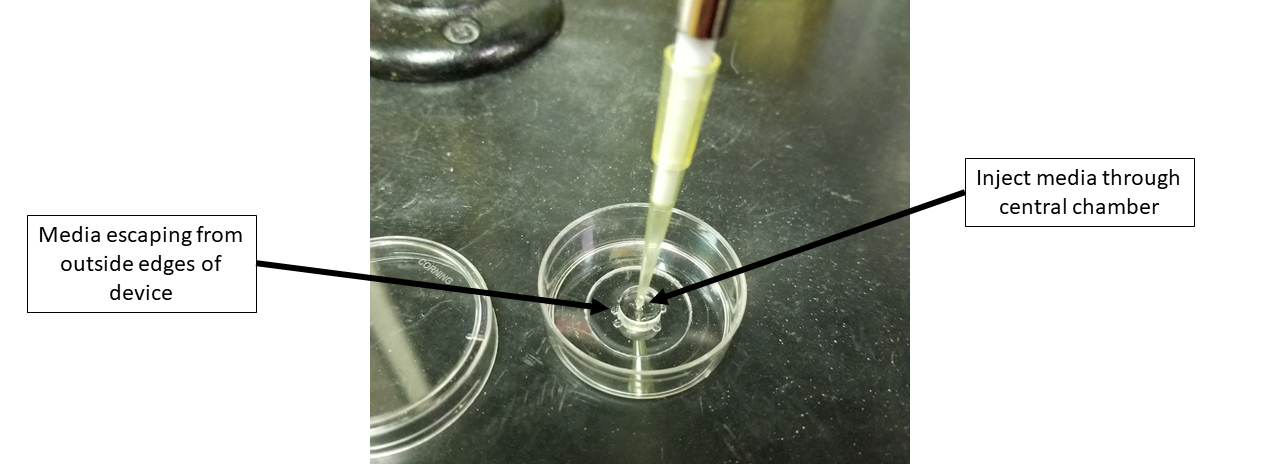


2.) Cover the top of the PDMS device with a bubble of media and place in a vacuum chamber for 10 minutes. Slowly release the vacuum to avoid sudden changes in pressure and disruption/contamination of device.


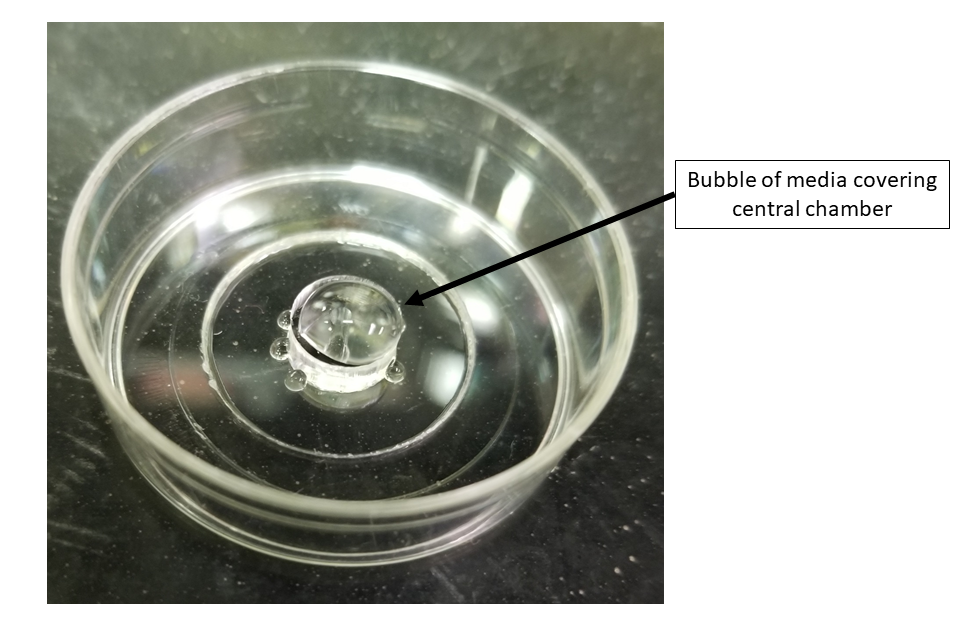


3.) Following vacuum, allow the device to equilibrate for an additional 10 minutes. If media didn’t prime completely in Step 1, as seen by media escaping from outside edges of PDMS device, then a second priming can be attempted here. Screen by microscopy to ensure all features are filled with media and any air bubbles are gone.


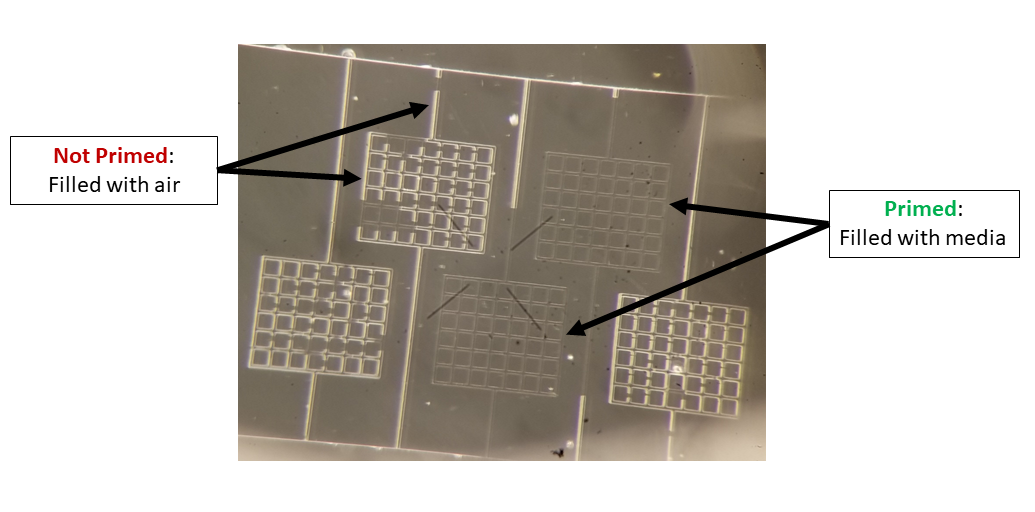


4.) Add media to the well (generally 2-3 mL) until it covers the top of the device.


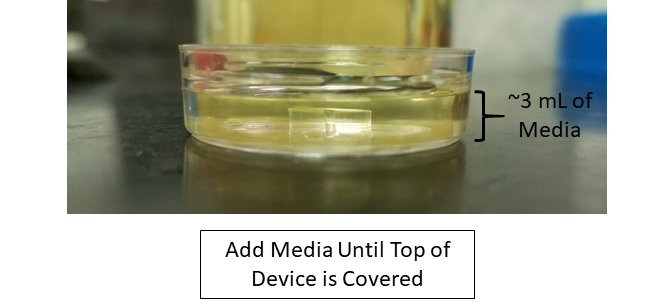


5.) Load the device with fungi of choice into the central port***. This step may need to be optimized for each fungal species. To start, we suggest adding between 1 to 5 µL of fungi (suggested starting concentration 2x10^7^ cfu/mL) to the central chamber.

6.) Allow to grow at optimal temperature overnight.

7.) After overnight incubation, remove the old media and wash the outside of the device to remove any external fungal growth.

8.) Add fresh media to the well to the same level as in step 4 (generally 2-3 mL).

9.) Select the field of view with features that are closest to the hyphal growth.

10.) Image.

*** Loading concentration and incubation time before imaging in steps #5-6 are suggestions and will likely need to be optimized for your fungus of choice.
