# Supplementary material for: Crowdsourced analysis of fungal growth and branching on microfluidic platforms
Source: PLoS One. 2021 Sep 29;16(9):e0257823. doi: 10.1371/journal.pone.0257823 (PMC8480888; doi:10.1371/journal.pone.0257823)
Supplement: Attachment — Submitted filename: Response to Reviewers_v4.docx [file pone.0257823.s010.docx]

We would like to thank the reviewers for reading our work, their positive responses to our revisions and for their continued constructive critiques. We have carefully revised the manuscript to reflect reviewer’s feedback and our point by point responses are highlighted below.

Minor issues and suggestions:
Lines 46-47: The authors made the suggested revision to omit the word ‘lifestyle’ here, but the abstract on page 1 of the manuscript is not revised. The authors should update the abstract on page 1 as well.

We apologize for the error and have updated the abstract in the submission system to match our updated manuscript abstract.

Lines 79-82: “Despite the clear advantages of microfluidics for the measurement of hyphal growth rates and branching patterns, many fungal research groups do not have access to have the engineering expertise needed to design and manufacture the devices or to the microscopy equipment needed to accurately record the results.”
There is a small error in this sentence that should be clarified.

We have updated this sentence. “The clear advantages of microfluidics for the measurement of hyphal growth rates and branching patterns are attracting many fungal research groups to use these technologies. However, where microfluidic devices have been used, current methodologies are often highly customized for each species and vary significantly between labs.”

Lines 98-99: In the methods section, the authors usually spell out the media recipe before abbreviating, except two instances. The authors should abbreviate the media recipe they used only after writing out the full name.

We have updated these lines to contain the full names of the media used, to match the rest of the methods section.

Line 148: Thank you to the authors for converting all units to cfu/mL. It greatly improves the clarity of the methods section. Line 148 is the only place in the methods that the concentration has not been converted to cfu/mL.

We have updated this line to correct the units to cfu/mL to match the unified units in the rest of the document.

Supplementary Figure 1 (Loading protocol): The units for the inoculum are in cells/mL. The concentration should be in cfu/mL to be consistent with the unit in the methods section of the paper.

We have updated the figure to now be cfu/mL, in line with the unified units in the manuscript.
